# Supplementary material for: Neurology-related protein biomarkers are associated with cognitive ability and brain volume in older age
Source: Nat Commun. 2020 Feb 10;11:800. doi: 10.1038/s41467-019-14161-7 (PMC7010796; doi:10.1038/s41467-019-14161-7)
Supplement: Supplementary file 1 — Supplementary Information [file 41467_2019_14161_MOESM1_ESM.pdf]

Neurology-related protein biomarkers are associated with cognitive ability and brain volume in older age

Harris *et al.*

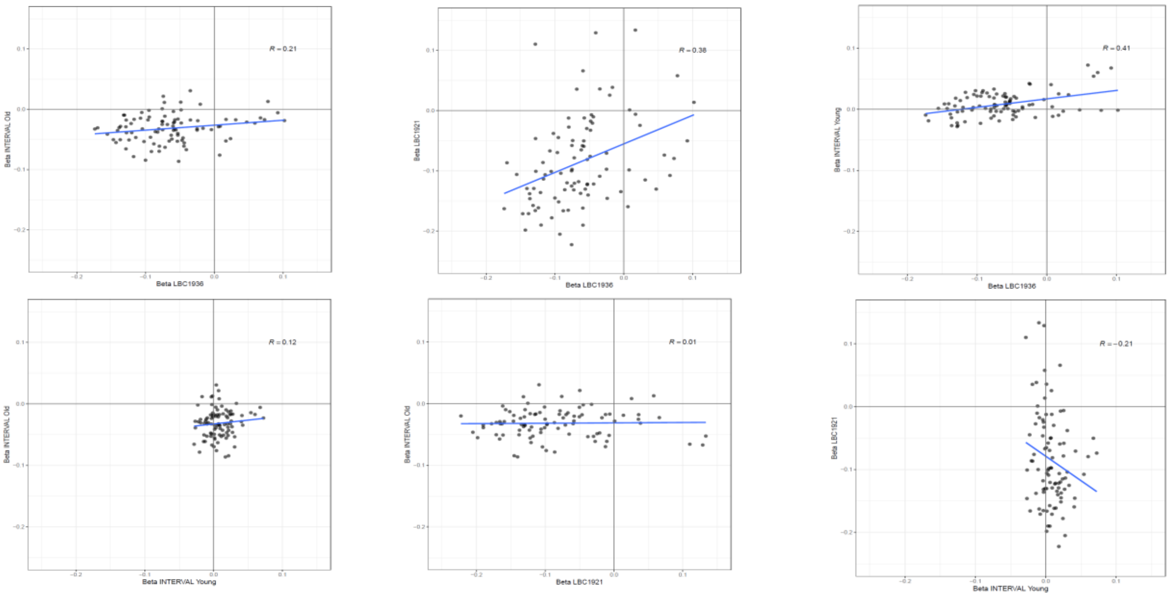

Supplementary Figure 1. Scattergraphs indicating the effect sizes (betas) for 90 proteins and protein PC1-PC3 for all four cohorts versus each other.

Supplementary Table 1. Component loadings for PC1-PC5 for LBC1936

	PC1	PC2	PC3	PC4	PC5
NMNAT1	0.14	0.6	0.02	-0.19	0.52
NRP2	0.67	0.17	0.1	0.13	-0.11
CADM3	0.78	-0.25	-0.17	-0.29	0.05
GDNF	0.49	0.03	0	0.18	-0.19
UNC5C	0.81	-0.25	-0.19	-0.03	0
VWC2	0.71	-0.25	-0.2	-0.17	-0.01
Siglec-9	0.46	0.18	0.07	0.37	0.06
CLM-6	0.69	0.01	-0.25	0.11	0.1
EZR	0.39	0.56	0.09	-0.07	0.5
SMOC2	0.69	-0.12	-0.05	-0.02	-0.08
NBL1	0.47	0.17	-0.18	-0.06	-0.1
EFNA4	0.84	-0.14	-0.3	0.06	0.03
SCARB2	0.79	-0.15	-0.33	0.09	0
NCAN	0.4	0.13	0.41	-0.26	0
PRTG	0.66	0.07	0.43	0.08	-0.1
ROBO2	0.66	-0.05	0.41	0.05	0.06
CRTAM	0.55	-0.08	-0.04	0.17	0.23
RGMA	0.68	-0.06	0.22	-0.27	-0.02
PLXNB3	0.35	0.79	-0.16	-0.12	-0.22
CPA2	0.3	0.05	-0.01	-0.03	-0.11
CD38	0.69	-0.04	-0.3	0.08	-0.01
SMPD1	0.38	0.12	0.11	0.34	-0.03
MSR1	0.62	-0.07	-0.09	0.38	0.04
Alpha-2-MRAP	0.36	0.65	-0.18	-0.06	-0.04
sFRP-3	0.44	0.05	0.15	0.05	0.08
EPHB6	0.83	-0.2	-0.17	-0.2	0.04
RGMB	0.86	-0.22	-0.06	-0.24	0
SIGLEC1	0.52	-0.12	-0.11	0.38	0.11
CNTN5	0.36	0.07	0.57	-0.15	0.07
ADAM22	0.77	-0.19	-0.11	-0.24	0.13
CLEC1B	0.2	0.81	-0.31	-0.09	-0.23
ADAM23	0.69	-0.13	0.07	-0.21	0.03
MATN3	0.47	0.02	0.22	-0.1	-0.24
RSPO1	0.71	0.07	-0.11	0.01	-0.17
HAGH	0.3	0.71	-0.21	-0.18	-0.04
LXN	0.35	0.53	-0.11	-0.07	-0.14
gal-8	0.25	0.76	-0.27	-0.11	-0.19
BCAN	0.5	0.07	0.37	-0.35	0.03
LAYN	0.84	-0.28	-0.22	-0.12	0
NEP	0.3	-0.02	0.06	0.45	-0.02
GDF-8	0.43	0.02	0.33	-0.18	0.09
THY-1	0.8	-0.2	-0.18	-0.04	0.11
WFIKKN1	0.3	0.16	0.12	-0.09	0.19
TMPRSS5	0.43	-0.03	0.34	-0.15	-0.11
CDH3	0.75	-0.2	-0.2	-0.12	0.1
GFR-alpha-1	0.79	-0.19	-0.19	0.01	-0.05
GM-CSF-R-alpha	0.29	0.04	0.08	0.22	0.12
Beta-NGF	0.61	0.05	-0.09	0.12	-0.23

SCARA5	0.81	-0.1	0	-0.06	-0.03
CD200	0.74	-0.02	0.19	-0.2	0.07
NTRK2	0.69	0.32	0.4	0	0
GZMA	0.35	0.51	-0.12	0.02	0.48
G-CSF	0.26	0.12	-0.14	0.24	0.04
DRAXIN	0.71	-0.17	-0.01	-0.05	0.06
SCARF2	0.77	-0.21	0.15	-0.13	-0.02
GDNFR-alpha-3	0.72	-0.06	0.19	-0.24	-0.05
PVR	0.57	0.12	0.21	0.31	-0.11
TNFRSF12A	0.74	-0.26	-0.19	0.03	-0.09
SKR3	0.81	-0.2	-0.27	0.02	-0.04
FLRT2	0.75	-0.1	0.11	0.01	0.01
CPM	0.52	0.18	0.09	0.35	0.06
CLEC10A	0.61	-0.09	-0.07	0.07	-0.02
GCP5	0.22	0.62	-0.1	-0.15	-0.16
BMP-4	0.48	-0.29	0.36	0.11	-0.1
FcRL2	0.41	-0.06	-0.01	0.19	0.2
MDGA1	0.44	-0.07	0.32	0.03	0.07
IL-5R-alpha	0.31	0	0.06	0.2	-0.22
PDGF-R-alpha	0.74	-0.09	0.21	0.12	-0.14
CTSC	0.4	0.46	0.02	0.17	-0.04
CDH6	0.71	-0.01	0.19	-0.01	-0.03
DDR1	0.76	0.01	0.27	0.12	-0.08
JAM-B	0.82	-0.16	-0.26	-0.21	0.06
CTSS	0.51	0.34	-0.24	0.05	0.08
N-CDase	0.26	0.15	0.17	0.28	0.14
NAAA	0.24	0.58	0.09	-0.08	0.41
N2DL-2	0.73	-0.14	-0.26	0.01	-0.02
PLXNB1	0.42	0.1	-0.09	0.06	-0.21
TNFRSF21	0.83	-0.15	-0.05	-0.09	0.02
CLM-1	0.53	-0.1	-0.33	0.08	0.05
SPOCK1	0.35	0.31	0.47	0.2	-0.18
IL12	0.51	-0.13	-0.25	0.14	0.25
Dkk-4	0.69	-0.1	-0.12	-0.06	-0.1
EDA2R	0.72	-0.28	-0.27	0.04	-0.06
LAT	0.16	0.76	-0.35	-0.1	-0.15
NTRK3	0.51	0.14	0.6	-0.19	-0.11
LAIR-2	0.31	-0.11	-0.14	0.04	0.18
MANF	0.38	0.52	-0.04	0.04	-0.23
TN-R	0.42	-0.03	0.31	-0.05	-0.05
CD200R1	0.49	0.07	0.14	0.2	0.12
Nr-CAM	0.64	0.12	0.34	0.18	0.06
KYNU	0.37	0.52	0.06	0.3	0.07

Supplementary Table 2. Component loadings for PC1-PC5 for INTERVAL-Old

	PC1	PC2	PC3	PC4	PC5
GCP5	0.35	0.42	0.16	-0.22	-0.01
Alpha-2-MRAP	0.45	0.51	0.07	-0.08	0.03
LAT	0.2	0.86	-0.02	-0.34	0.03
gal-8	0.25	0.74	-0.01	-0.29	0.07
PLXNB3	0.38	0.73	0.1	-0.33	0.06
CLEC1B	0.32	0.81	-0.04	-0.27	0.02
LXN	0.45	0.39	0.05	0.01	-0.11
MANF	0.23	0.78	0.03	-0.28	0.06
KYNU	0.43	0.5	-0.06	0.27	-0.01
GZMA	0.52	0.19	-0.13	0.21	0.19
NAAA	0.34	0.14	0.2	0.12	-0.08
NMNAT1	0.27	0.3	-0.09	0.18	-0.12
EZR	0.42	0.27	-0.13	0.15	-0.03
N-CDase	0.28	0.16	0.07	0.31	-0.16
NEP	0.27	0.18	0.06	0.38	-0.24
PVR	0.58	0.07	0.2	0.22	-0.19
CPM	0.46	0.3	-0.03	0.37	-0.15
CPA2	0.33	0.03	0.16	0.09	-0.1
IL-5R-alpha	0.31	0.08	0	0.08	0.04
GDNF	0.49	0.11	0.03	0.06	-0.2
PLXNB1	0.39	0.12	0.04	0.02	0.04
NBL1	0.28	0.14	-0.14	-0.12	0.06
CTSS	0.37	0.25	-0.09	0.01	-0.17
CLEC10A	0.56	-0.05	-0.03	0.07	-0.06
RGMA	0.64	-0.24	0.16	-0.12	-0.11
ADAM-23	0.62	-0.13	0.04	-0.12	-0.12
PDGF-R-alpha	0.71	-0.09	0.19	0.03	-0.01
DDR1	0.82	-0.02	0.15	0.08	0
CDH6	0.64	-0.16	0.14	-0.03	-0.01
SCARF2	0.73	-0.27	0.11	-0.14	0.02
FLRT2	0.74	-0.15	0.05	-0.01	-0.09
DRAXIN	0.6	-0.14	-0.08	-0.09	0.13
CD200	0.74	-0.21	0.21	-0.16	0.11
GDNFR-alpha-3	0.7	-0.2	0.2	-0.17	-0.02
CLM-6	0.64	0.03	-0.29	0.08	0.08
CLM-1	0.49	0.06	-0.31	0.05	0.06
Beta-NGF	0.51	0.1	0	-0.04	-0.09
N2DL-2	0.7	-0.08	-0.22	-0.05	-0.05
THY-1	0.74	-0.1	-0.18	-0.07	0.03
ADAM-22	0.72	-0.18	-0.18	-0.18	0.02
RGMB	0.83	-0.22	-0.06	-0.21	-0.11
JAM-B	0.79	-0.26	-0.21	-0.18	-0.11
CADM3	0.74	-0.25	-0.05	-0.3	0.09
EPHB6	0.81	-0.2	-0.17	-0.23	0.04
LAYN	0.78	-0.26	-0.17	-0.21	-0.04
VWC2	0.62	-0.14	-0.34	-0.12	0.02
CDH3	0.66	-0.26	-0.22	-0.19	0.03
UNC5C	0.74	-0.14	-0.27	-0.11	0.01

TNFRSF21	0.81	-0.16	-0.1	-0.08	0.07
RSPO1	0.67	0.27	0.03	-0.1	-0.09
Dkk-4	0.57	0.06	-0.02	-0.03	-0.11
CD38	0.63	0.03	-0.23	0.06	-0.13
SMOC2	0.6	0.02	-0.05	0.01	-0.33
SKR3	0.79	-0.04	-0.19	0	-0.16
EDA2R	0.65	-0.16	-0.18	-0.09	-0.18
TNFRSF12A	0.65	-0.04	-0.12	0.03	-0.24
EFNA4	0.83	-0.01	-0.27	0.05	-0.07
SCARB2	0.73	-0.07	-0.32	0.1	0.01
GFR-alpha-1	0.76	-0.02	-0.19	0.03	-0.05
SCARA5	0.75	0.01	-0.09	0.05	-0.16
G-CSF	0.26	0.13	-0.15	0.11	0.09
LAIR-2	0.25	0.03	-0.17	0.04	0.18
FcRL2	0.45	0.01	-0.14	0.13	0.22
MSR1	0.56	0.13	-0.21	0.3	0.2
SIGLEC1	0.46	0.07	-0.27	0.29	0.33
CRTAM	0.5	-0.06	-0.18	0.14	0.36
IL12	0.43	-0.01	-0.35	0.09	0.24
GM-CSF-R-alpha	0.27	0.01	-0.06	0.18	0.18
Siglec-9	0.4	0.1	0	0.27	0.29
CD200R1	0.51	-0.01	0.01	0.23	0.22
SMPD1	0.39	0.12	0.13	0.29	-0.19
CTSC	0.38	0.28	0.14	0.17	-0.08
NRP2	0.54	0.08	0.15	0.12	0.2
ROBO2	0.72	-0.12	0.29	0.12	0.09
Nr-CAM	0.69	0.08	0.32	0.15	0.14
SPOCK1	0.46	0.12	0.49	0.16	0.08
PRTG	0.62	-0.05	0.37	0.09	-0.08
NTRK2	0.78	0.13	0.29	0.16	-0.02
NTRK3	0.66	-0.1	0.5	-0.01	-0.08
sFRP-3	0.54	0.16	0.01	0.08	0.11
WFIKKN1	0.38	0	0.01	0.17	0.17
TMPRSS5	0.46	-0.14	0.33	-0.09	0.1
MATN3	0.54	-0.02	0.18	-0.18	-0.11
NCAN	0.42	-0.1	0.46	-0.15	0.33
BCAN	0.5	-0.16	0.41	-0.25	0.26
TN-R	0.42	0.01	0.13	-0.05	0.17
GDF-8	0.47	-0.11	0.14	0.05	-0.24
BMP-4	0.33	-0.34	0.21	0.12	-0.12
CNTN5	0.42	-0.13	0.4	-0.05	0.12
MDGA1	0.4	-0.13	0.22	0.12	0.11
IL12	0.439	-0.11	0.236	-0.23	0.341

Supplementary Table 3. Component loadings for PC1-PC5 for INTERVAL-Young

	PC1	PC2	PC3	PC4	PC5
GCP5	0.35	0.42	0.16	-0.22	-0.01
Alpha-2-MRAP	0.45	0.51	0.07	-0.08	0.03
LAT	0.2	0.86	-0.02	-0.34	0.03
gal-8	0.25	0.74	-0.01	-0.29	0.07
PLXNB3	0.38	0.73	0.1	-0.33	0.06
CLEC1B	0.32	0.81	-0.04	-0.27	0.02
LXN	0.45	0.39	0.05	0.01	-0.11
MANF	0.23	0.78	0.03	-0.28	0.06
KYNU	0.43	0.5	-0.06	0.27	-0.01
GZMA	0.52	0.19	-0.13	0.21	0.19
NAAA	0.34	0.14	0.2	0.12	-0.08
NMNAT1	0.27	0.3	-0.09	0.18	-0.12
EZR	0.42	0.27	-0.13	0.15	-0.03
N-CDase	0.28	0.16	0.07	0.31	-0.16
NEP	0.27	0.18	0.06	0.38	-0.24
PVR	0.58	0.07	0.2	0.22	-0.19
CPM	0.46	0.3	-0.03	0.37	-0.15
CPA2	0.33	0.03	0.16	0.09	-0.1
IL-5R-alpha	0.31	0.08	0	0.08	0.04
GDNF	0.49	0.11	0.03	0.06	-0.2
PLXNB1	0.39	0.12	0.04	0.02	0.04
NBL1	0.28	0.14	-0.14	-0.12	0.06
CTSS	0.37	0.25	-0.09	0.01	-0.17
CLEC10A	0.56	-0.05	-0.03	0.07	-0.06
RGMA	0.64	-0.24	0.16	-0.12	-0.11
ADAM-23	0.62	-0.13	0.04	-0.12	-0.12
PDGF-R-alpha	0.71	-0.09	0.19	0.03	-0.01
DDR1	0.82	-0.02	0.15	0.08	0
CDH6	0.64	-0.16	0.14	-0.03	-0.01
SCARF2	0.73	-0.27	0.11	-0.14	0.02
FLRT2	0.74	-0.15	0.05	-0.01	-0.09
DRAXIN	0.6	-0.14	-0.08	-0.09	0.13
CD200	0.74	-0.21	0.21	-0.16	0.11
GDNFR-alpha-3	0.7	-0.2	0.2	-0.17	-0.02
CLM-6	0.64	0.03	-0.29	0.08	0.08
CLM-1	0.49	0.06	-0.31	0.05	0.06
Beta-NGF	0.51	0.1	0	-0.04	-0.09
N2DL-2	0.7	-0.08	-0.22	-0.05	-0.05
THY-1	0.74	-0.1	-0.18	-0.07	0.03
ADAM-22	0.72	-0.18	-0.18	-0.18	0.02
RGMB	0.83	-0.22	-0.06	-0.21	-0.11
JAM-B	0.79	-0.26	-0.21	-0.18	-0.11
CADM3	0.74	-0.25	-0.05	-0.3	0.09
EPHB6	0.81	-0.2	-0.17	-0.23	0.04
LAYN	0.78	-0.26	-0.17	-0.21	-0.04
VWC2	0.62	-0.14	-0.34	-0.12	0.02
CDH3	0.66	-0.26	-0.22	-0.19	0.03
UNC5C	0.74	-0.14	-0.27	-0.11	0.01

TNFRSF21	0.81	-0.16	-0.1	-0.08	0.07
RSPO1	0.67	0.27	0.03	-0.1	-0.09
Dkk-4	0.57	0.06	-0.02	-0.03	-0.11
CD38	0.63	0.03	-0.23	0.06	-0.13
SMOC2	0.6	0.02	-0.05	0.01	-0.33
SKR3	0.79	-0.04	-0.19	0	-0.16
EDA2R	0.65	-0.16	-0.18	-0.09	-0.18
TNFRSF12A	0.65	-0.04	-0.12	0.03	-0.24
EFNA4	0.83	-0.01	-0.27	0.05	-0.07
SCARB2	0.73	-0.07	-0.32	0.1	0.01
GFR-alpha-1	0.76	-0.02	-0.19	0.03	-0.05
SCARA5	0.75	0.01	-0.09	0.05	-0.16
G-CSF	0.26	0.13	-0.15	0.11	0.09
LAIR-2	0.25	0.03	-0.17	0.04	0.18
FcRL2	0.45	0.01	-0.14	0.13	0.22
MSR1	0.56	0.13	-0.21	0.3	0.2
SIGLEC1	0.46	0.07	-0.27	0.29	0.33
CRTAM	0.5	-0.06	-0.18	0.14	0.36
IL12	0.43	-0.01	-0.35	0.09	0.24
GM-CSF-R-alpha	0.27	0.01	-0.06	0.18	0.18
Siglec-9	0.4	0.1	0	0.27	0.29
CD200R1	0.51	-0.01	0.01	0.23	0.22
SMPD1	0.39	0.12	0.13	0.29	-0.19
CTSC	0.38	0.28	0.14	0.17	-0.08
NRP2	0.54	0.08	0.15	0.12	0.2
ROBO2	0.72	-0.12	0.29	0.12	0.09
Nr-CAM	0.69	0.08	0.32	0.15	0.14
SPOCK1	0.46	0.12	0.49	0.16	0.08
PRTG	0.62	-0.05	0.37	0.09	-0.08
NTRK2	0.78	0.13	0.29	0.16	-0.02
NTRK3	0.66	-0.1	0.5	-0.01	-0.08
sFRP-3	0.54	0.16	0.01	0.08	0.11
WFIKKN1	0.38	0	0.01	0.17	0.17
TMPRSS5	0.46	-0.14	0.33	-0.09	0.1
MATN3	0.54	-0.02	0.18	-0.18	-0.11
NCAN	0.42	-0.1	0.46	-0.15	0.33
BCAN	0.5	-0.16	0.41	-0.25	0.26
TN-R	0.42	0.01	0.13	-0.05	0.17
GDF-8	0.47	-0.11	0.14	0.05	-0.24
BMP-4	0.33	-0.34	0.21	0.12	-0.12
CNTN5	0.42	-0.13	0.4	-0.05	0.12
MDGA1	0.4	-0.13	0.22	0.12	0.11
IL12	0.43		-0.209	-0.283	0.214

Supplementary Table 4. Component loadings for PC1-PC5 for LBC1921

	PC1	PC2	PC3	PC4	PC5
NMNAT1	0.11	0.51	-0.03	0.34	0.36
NRP2	0.55	0.11	0.17	0.09	-0.24
CADM3	0.83	-0.22	-0.15	-0.24	0.07
GDNF	0.45	0.18	0.15	-0.03	0.05
UNC5C	0.84	-0.17	-0.25	-0.07	0.01
VWC2	0.71	-0.23	-0.19	-0.13	-0.02
Siglec-9	0.38	0.1	0.17	0.38	-0.2
CLM-6	0.77	-0.02	-0.23	0.2	-0.1
EZR	0.47	0.25	0.01	0.27	0.3
SMOC2	0.76	-0.09	-0.05	-0.21	-0.05
NBL1	0.35	0.21	-0.09	-0.14	-0.03
EFNA4	0.87	-0.14	-0.31	0.06	0.09
SCARB2	0.85	-0.08	-0.32	-0.03	0.05
NCAN	0.29	-0.01	0.52	-0.24	-0.03
PRTG	0.59	-0.03	0.41	0.05	-0.22
ROBO2	0.62	-0.2	0.44	0.1	-0.12
CRTAM	0.6	-0.04	-0.1	0.3	-0.16
RGMA	0.66	-0.13	0.15	-0.36	0.07
PLXNB3	0.35	0.83	-0.02	-0.19	-0.08
CPA2	0.29	-0.11	0.2	-0.17	0.12
CD38	0.73	0.03	-0.35	-0.01	0.13
SMPD1	0.26	0.17	0.34	0.03	0.36
MSR1	0.57	-0.1	-0.08	0.15	-0.16
Alpha-2-MRAP	0.36	0.66	-0.06	-0.13	-0.16
sFRP-3	0.46	0.09	0.26	0.33	-0.1
EPHB6	0.87	-0.14	-0.16	-0.12	0.05
RGMB	0.87	-0.19	-0.17	-0.2	0.04
SIGLEC1	0.49	-0.02	-0.13	0.44	-0.18
CNTN5	0.33	-0.13	0.46	0.19	-0.26
ADAM-22	0.85	-0.24	-0.19	-0.05	0.11
CLEC1B	0.29	0.85	-0.16	-0.11	-0.17
ADAM-23	0.69	-0.1	0.14	-0.11	0.08
MATN3	0.42	0.12	0.21	-0.38	-0.01
RSPO1	0.8	0.19	-0.09	-0.1	-0.16
HAGH	0.27	0.75	-0.12	-0.05	-0.09
LXN	0.21	0.5	0.08	0.05	-0.14
gal-8	0.35	0.82	-0.1	-0.16	-0.07
BCAN	0.51	-0.12	0.35	-0.26	-0.03
LAYN	0.87	-0.25	-0.24	-0.06	0.03
NEP	0.36	-0.02	0.21	0.13	0.21
GDF-8	0.47	-0.03	0.28	-0.17	0.15
THY-1	0.85	-0.14	-0.21	0.05	0.08
WFIKKN1	0.2	0.09	0.17	0.05	0.37
TMPRSS5	0.4	-0.16	0.28	-0.24	-0.11
CDH3	0.79	-0.19	-0.22	0.04	0.1
GFR-alpha-1	0.77	-0.17	-0.23	-0.12	-0.16
GM-CSF-R-alpha	0.26	0.01	0.05	0.22	-0.16
Beta-NGF	0.6	0.2	-0.11	0.02	-0.06

SCARA5	0.83	0.01	-0.11	-0.13	0.12
CD200	0.78	-0.08	0.19	0.01	0.01
NTRK2	0.66	0.09	0.5	0.03	0.04
GZMA	0.42	0.28	-0.22	0.33	0.13
G-CSF	0.21	0.06	-0.15	0.02	-0.23
DRAXIN	0.74	-0.16	-0.13	-0.05	-0.12
SCARF2	0.83	-0.13	0.15	-0.01	-0.1
GDNFR-alpha-3	0.77	-0.14	0.15	-0.21	0.02
PVR	0.6	0.08	0.29	0.09	0.04
TNFRSF12A	0.81	-0.05	-0.18	-0.1	0.15
SKR3	0.89	-0.06	-0.21	-0.04	0.04
FLRT2	0.77	-0.09	0.06	0.07	-0.08
CPM	0.42	-0.01	0.33	0.02	0.45
CLEC10A	0.67	0.01	0	0.04	0.03
GCP5	0.25	0.58	-0.12	-0.17	-0.06
BMP-4	0.52	-0.19	0.4	0.08	-0.02
FcRL2	0.47	-0.05	-0.02	0.4	0.02
MDGA1	0.45	-0.13	0.35	0.06	-0.13
IL-5R-alpha	0.24	-0.06	-0.03	-0.08	-0.22
PDGF-R-alpha	0.69	0.01	0.29	-0.01	0.05
CTSC	0.28	0.49	0.22	0.17	0.03
CDH6	0.72	-0.04	0.19	0.11	-0.13
DDR1	0.78	0	0.32	0.09	-0.02
JAM-B	0.87	-0.09	-0.28	-0.11	0.07
CTSS	0.53	0.2	-0.12	0.15	0.06
N-CDase	0.15	-0.07	0.24	-0.02	0.39
NAAA	0.1	0.37	0.22	0.05	0.53
N2DL-2	0.82	-0.03	-0.24	0.12	-0.07
PLXNB1	0.38	0.27	-0.08	-0.18	0.15
TNFRSF21	0.86	-0.13	-0.03	-0.04	0.02
CLM-1	0.61	0.04	-0.33	0.25	0.02
SPOCK1	0.26	0.19	0.55	-0.04	-0.05
IL12	0.5	-0.14	-0.39	0.31	0.13
Dkk-4	0.72	-0.12	-0.12	-0.1	-0.02
EDA2R	0.85	-0.11	-0.26	-0.1	-0.05
LAT	0.19	0.84	-0.2	-0.09	-0.08
NTRK3	0.51	0.02	0.65	-0.03	-0.18
LAIR-2	0.37	0.01	-0.33	0.18	0.07
MANF	0.35	0.68	0.02	-0.01	-0.08
TN-R	0.37	0.04	0.26	-0.06	-0.07
CD200R1	0.45	-0.03	0.21	0.45	-0.02
Nr-CAM	0.62	0.02	0.41	0.17	0.05
KYNU	0.36	0.54	0.11	0.02	0.18

Supplementary Table 5. Pearson correlations between normalised protein expression levels in LBC1936 for proteins used in the regional distribution of protein-cortical associations
All correlations significant at p<0.01.

	EDA2R	PVR	SKR3	MSR1	GFR-alpha-1
EDA2R	1	0.375	0.758	0.494	0.653
PVR	0.375	1	0.396	0.403	0.408
SKR3	0.758	0.396	1	0.545	0.705
MSR1	0.494	0.403	0.545	1	0.489
GFR-alpha-1	0.653	0.408	0.705	0.489	1

Supplementary Table 6. Percentage of samples below the lower limit of detection for LBC and INTERVAL for each protein

Protein	LBC	INTERVAL
ADAM 22	0%	0%
ADAM 23	0%	0%
Alpha-2-MRAP	0%	0%
BCAN	0%	0%
BDNF	NA	17%
Beta-NGF	1%	0%
BMP-4	0%	0%
CADM3	0%	0%
CD200	0%	0%
CD200R1	0%	0%
CD38	0%	0%
CDH3	0%	0%
CDH6	0%	0%
CLEC10A	0%	0%
CLEC1B	0%	0%
CLM-1	0%	0%
CLM-6	0%	0%
CNTN5	0%	0%
CPA2	0%	0%
CPM	0%	0%
CRTAM	0%	0%
CTSC	0%	0%
CTSS	0%	0%
DDR1	0%	0%
Dkk-4	0%	0%
DRAXIN	0%	0%
EDA2R	0%	0%
EFNA4	0%	0%
EPHB6	0%	0%
EZR	0%	0%
FcRL2	0%	0%
FLRT2	0%	0%
gal-8	0%	0%
GCP5	0%	0%
G-CSF	0%	7%
GDF-8	0%	0%
GDNF	2%	9%
GDNFR-alpha-3	0%	0%
GFR-alpha-1	0%	0%
GM-CSF-R-alpha	0%	0%
GZMA	0%	0%
HAGH	98%	23%
IL12	0%	0%
IL-5R-alpha	0%	0%
JAM-B	0%	0%
KYNU	0%	0%
LAIR-2	3%	3%

LAT	2%	4%
LAYN	0%	0%
LXN	2%	0%
MANF	0%	0%
MAPT	100%	NA
MATN3	0%	0%
MDGA1	0%	0%
MSR1	0%	0%
N2DL-2	0%	0%
NAAA	0%	0%
NBL1	0%	0%
NCAN	0%	0%
N-CDase	0%	0%
NEP	0%	0%
NMNAT1	4%	0%
Nr-CAM	0%	0%
NRP2	0%	0%
NTRK2	0%	0%
NTRK3	0%	0%
PDGF-R-alpha	0%	0%
PLXNB1	0%	0%
PLXNB3	0%	0%
PRTG	0%	0%
PVR	0%	0%
RGMA	0%	0%
RGMB	0%	0%
ROBO2	0%	0%
RSPO1	0%	0%
SCARA5	0%	0%
SCARB2	0%	0%
SCARF2	0%	0%
sFRP-3	0%	0%
SIGLEC1	0%	0%
Siglec-9	0%	0%
SKR3	0%	0%
SMOC2	0%	0%
SMPD1	0%	0%
SPOCK1	0%	0%
THY 1	0%	0%
TMPRSS5	0%	0%
TNFRSF12A	0%	0%
TNFRSF21	0%	0%
TN-R	0%	0%
UNC5C	0%	0%
VWC2	0%	0%
WFIKKN1	0%	0%
