# Supplementary material for: Neurology-related protein biomarkers are associated with cognitive ability and brain volume in older age
Source: Nat Commun. 2020 Feb 10;11:800. doi: 10.1038/s41467-019-14161-7 (PMC7010796; doi:10.1038/s41467-019-14161-7)
Supplement: Supplementary file 11 — Description of Additional Supplementary Files [file 41467_2019_14161_MOESM11_ESM.docx]

**Description of Additional Supplementary Files**

File Name: Supplementary Data 1

Description: Proportion of variance explained by the first 17 components from a PCA on

OLINK proteins across cohorts, and their factor congruence. Note: INTERVAL y and o denote the young and old INTERVAL cohorts respectively. Congruence denotes the coefficient of factor congruence.

File Name: Supplementary Data 2

Description: Association of proteins and protein-PC1-PC3 with general fluid cognitive

ability in LBC1936, INTERVAL-Old, INTERVAL-Young and LBC1921. Meta-analysis of LBC1936 and INTERVAL-Old is also shown. Corrected for age and sex.

File Name: Supplementary Data 3

Description: Association of proteins and protein-PC1-PC3 with general fluid cognitive ability in LBC1936, INTERVAL-Old and INTERVAL-Young. Meta-analysis of LBC1936 and INTERVALOld is also shown. Corrected for age, sex, smoking status and antihypertension medication use.

File Name: Supplementary Data 4

Description: Association of proteins and protein-PC1-PC3 with brain variables in LBC1936. Corrected for age and sex.

File Name: Supplementary Data 5

Description: Association of proteins and protein-PC1-PC3 with brain variables in LBC1936. Corrected for age, sex, smoking status and antihypertension medication use.

File Name: Supplementary Data 6

Description: Mediation anlaysis in LBC1936 separated by brain variable.

File Name: Supplementary Data 7

Description: Mediation of association between protein-PC1 and proteins and general fluid cognitive ability by total brain volume in LBC1936. Corrected for age, sex, smoking status and antihypertension medication use.

File Name: Supplementary Data 8

Description: Mediation of association between protein-PC1 and proteins and general

cognitive fluid ability by MRI brain variables in LBC1936: grey matter volume, normal appearing white matter volume, white matter hyperintensity volume, perivascular spaces, general fractional anisotropy and general mean diffusivity. Corrected for age, sex, smoking status and antihypertension medication use.
