# Supplementary material for: Neurology-related protein biomarkers are associated with cognitive ability and brain volume in older age
Source: Nat Commun. 2020 Feb 10;11:800. doi: 10.1038/s41467-019-14161-7 (PMC7010796; doi:10.1038/s41467-019-14161-7)
Supplement: Supplementary file 12 — Reporting Summary [file 41467_2019_14161_MOESM12_ESM.pdf]

nature research

Corresponding author(s): Sarah E Harris

Last updated by author(s): Sep 12, 2019

Reporting Summary

Nature Research wishes to improve the reproducibility of the work that we publish. This form provides structure for consistency and transparency in reporting. For further information on Nature Research policies, see Authors & Referees and the Editorial Policy Checklist .

Statistics

For all statistical analyses, confirm that the following items are present in the figure legend, table legend, main text, or Methods section.

n/a	Confirmed	
☐	☒	The exact sample size (*n*) for each experimental group/condition, given as a discrete number and unit of measurement
☐	☒	A statement on whether measurements were taken from distinct samples or whether the same sample was measured repeatedly
☐	☒	The statistical test(s) used AND whether they are one- or two-sided<br>*Only common tests should be described solely by name; describe more complex techniques in the Methods section.*
☐	☒	A description of all covariates tested
☐	☒	A description of any assumptions or corrections, such as tests of normality and adjustment for multiple comparisons
☐	☒	A full description of the statistical parameters including central tendency (e.g. means) or other basic estimates (e.g. regression coefficient) AND variation (e.g. standard deviation) or associated estimates of uncertainty (e.g. confidence intervals)
☐	☒	For null hypothesis testing, the test statistic (e.g. *F*, *t*, *r*) with confidence intervals, effect sizes, degrees of freedom and *P* value noted<br>*Give P values as exact values whenever suitable.*
☒	☐	For Bayesian analysis, information on the choice of priors and Markov chain Monte Carlo settings
☒	☐	For hierarchical and complex designs, identification of the appropriate level for tests and full reporting of outcomes
☐	☒	Estimates of effect sizes (e.g. Cohen's *d*, Pearson's *r*), indicating how they were calculated

Our web collection on statistics for biologists contains articles on many of the points above.

Software and code

Policy information about availability of computer code

Data collection	N/A
Data analysis	R3.6.0, SPSS v24, METAL, SurfStat toolbox. Code available from corresponding author on request.

For manuscripts utilizing custom algorithms or software that are central to the research but not yet described in published literature, software must be made available to editors/reviewers. We strongly encourage code deposition in a community repository (e.g. GitHub). See the Nature Research guidelines for submitting code & software for further information.

Data

Policy information about availability of data

All manuscripts must include a data availability statement. This statement should provide the following information, where applicable:

- Accession codes, unique identifiers, or web links for publicly available datasets
- A list of figures that have associated raw data
- A description of any restrictions on data availability

Data supporting the findings of this manuscript are available from the corresponding author upon reasonable request.

Field-specific reporting

Please select the one below that is the best fit for your research. If you are not sure, read the appropriate sections before making your selection.

☐ Life sciences ☒ Behavioural & social sciences ☐ Ecological, evolutionary & environmental sciences

For a reference copy of the document with all sections, see nature.com/documents/nr-reporting-summary-flat.pdf

Behavioural & social sciences study design

All studies must disclose on these points even when the disclosure is negative.

Study description	cross-sectional cohort study
Research sample	The Lothian Birth Cohorts of 1921 and 1936 (Taylor AM, Pattie A, Deary IJ. Cohort Profile Update: The Lothian Birth Cohorts of 1921 and 1936. Int J Epidemiol 2018; 47: 1042-1042r). INTERVAL (Moore C, Sambrook J, Walker M, Tolkien Z, Kaptoge S, Allen D et al. The INTERVAL trial to determine whether intervals between blood donations can be safely and acceptably decreased to optimise blood supply: study protocol for a randomised controlled trial. Trials 2014; 15: 363.).
Sampling strategy	Convenience sample. All samples with relevant data were used.
Data collection	Data collection is described in the published study protocols (see research sample).
Timing	Timing of data collection is described in the published study protocols (see research sample).
Data exclusions	No data were excluded from the analyses.
Non-participation	All participants with relevant data who took part in the relevant wave of data collection were used.
Randomization	Participants were not allocated into randomized groups.

Reporting for specific materials, systems and methods

We require information from authors about some types of materials, experimental systems and methods used in many studies. Here, indicate whether each material, system or method listed is relevant to your study. If you are not sure if a list item applies to your research, read the appropriate section before selecting a response.

Materials & experimental systems

n/a	Involved in the study	
☒	☐	Antibodies
☒	☐	Eukaryotic cell lines
☒	☐	Palaeontology
☒	☐	Animals and other organisms
☐	☒	Human research participants
☒	☐	Clinical data

Methods

n/a	Involved in the study	
☒	☐	ChIP-seq
☒	☐	Flow cytometry
☐	☒	MRI-based neuroimaging

Human research participants

Policy information about studies involving human research participants

Population characteristics	See above
Recruitment	Recruitment is described in the study protocols. See above.
Ethics oversight	Ethics permission for the LBC1936 was obtained from the Scotland A Research Ethics Committee (07/MRE00/58). Ethics permission for the LBC1921 was obtained from the Lothian Research Ethics Committee (1702/98/4/183). The INTERVAL trial has received ethics committee approval from the National Research Ethics Service Committee East of England- (REC 11/EE/0538 All persons gave their informed consent prior to their inclusion in the study.

Note that full information on the approval of the study protocol must also be provided in the manuscript.

Magnetic resonance imaging

Experimental design

Design type	Global brain structural parameters were acquired and derived according to a previously published study protocol (Wardlaw JM, Bastin ME, Valdés Hernández MC, Maniega SM, Royle NA, Morris Z et al. Brain aging, cognition in youth and old age and vascular disease in the Lothian Birth Cohort 1936: rationale, design and methodology of the imaging protocol. Int J Stroke 2011; 6: 547–59.)
Design specifications	See above

Behavioral performance measures: See above

Acquisition

Imaging type(s): Structural and diffusion Magnetic Resonance Imaging; see above

Field strength: 1.5T

Sequence & imaging parameters: T2W, T2*W and FLAIR axial scans, a high-resolution T1W volume sequence acquired in the coronal plane, axial T1W fast-spoiled gradient echo (FSPGR) sequences with 2° and 12° flip angles for quantitative T1-mapping, two standard spin echo sequences acquired with and without a MT pulse applied 1kHz from the water resonance frequency for MT-MRI, and finally a DT-MRI protocol consisting of seven T2W and sets of diffusion-weighted (b=1000s/mm2) axial single-shot spin-echo echo-planar (EP) volumes acquired with diffusion gradients applied in 64 noncollinear directions.

Area of acquisition: Whole brain

Diffusion MRI: [x] Used [ ] Not used

Parameters: See published protocol described above.

Preprocessing

Preprocessing software: FSL (v 5; https://fsl.fmrib.ox.ac.uk/fsl/fslwiki) and Tractor (v 3; https://www.tractor-mri.org.uk)

Normalization: N/A

Normalization template: N/A

Noise and artifact removal: Provided as part of FSL and Tractor

Volume censoring: N/A

Statistical modeling & inference

Model type and settings: Global structural brain parameters were derived and used in linear regressions to quantify the magnitude and Bonferroni-corrected significance of their associations with 91 neurology-related proteins. They were also used as multiple correlated mediators in associations between the proteins and cognitive function. Based on these mediation models, FDR-corrected associations for cortical grey matter volume at the vertex level were ascertained for the proteins whose associations with cognitive function were most strongly mediated by brain parameters (global grey matter volume consistently accounted for a large proportion of the mediation effect).

Effect(s) tested: Covariates were associations of no interest; effects of interest were coefficients for the association between brain and protein in all cases.

Specify type of analysis: [x] Whole brain [ ] ROI-based [ ] Both

Statistic type for inference (See Eklund et al. 2016): We note that Eklund et al 2016 refers to inferences using cluster-wise statistics on fMRI data, which are not applicable for the present analyses. For the abovementioned vertex-wise cortical grey matter analyses, we calculated the t-value and FDR-corrected significance at each of >327,000 vertices across the entire pial surface.

Correction: False Discovery Rate

Models & analysis

n/a	Involved in the study
[x]	[ ] Functional and/or effective connectivity
[x]	[ ] Graph analysis
[ ]	[x] Multivariate modeling or predictive analysis

Multivariate modeling and predictive analysis: Linear regression models were used to test the associations of each of the 91 neurology-related protein biomarkers with: total brain, grey matter, normal appearing white matter and WMH volumes; and PVS, gFA and gMD. Mediation analysis in a structural equation modelling framework to identify if the significant (Bonferroni-corrected) protein-cognitive ability associations were mediated by the brain MRI variables. Brain cortical volumetric analyses were conducted using the SurfStat toolbox, described above.
